# Thrombin generation to evaluate the complex hemostatic balance of hemophilia A plasma containing direct oral anticoagulant and supplemented by factor VIII

**DOI:** 10.1016/j.rpth.2024.102576

**Published:** 2024-09-23

**Authors:** Sylvain Lamoine, Vincent Jury, Virginie Fourneyron, Jonathan Douxfils, Dorian Teissandier, Laurie Talon, Thomas Sinegre, Aurélien Lebreton

**Affiliations:** 1 Hematology Department, University Hospital of Clermont-Ferrand, Clermont-Ferrand, France; 2 Department of Pharmacy, Clinical Pharmacology and Toxicology Research Unit, Namur Research Institute for Life Sciences (NARILIS), University of Namur, Namur, Belgium; 3 QUALIresearch, QUALIblood s.a., Namur, Belgium; 4 Emergency Department, University Hospital of Clermont-Ferrand, Clermont-Ferrand, France; 5 Université Clermont Auvergne, INRAE, UNH, Clermont-Ferrand, France

**Keywords:** direct oral anticoagulant, emicizumab, hemophilia A, monitoring, thrombin

## Abstract

**Background:**

The incidence of cardiovascular diseases is increasing in persons with hemophilia A (HA). Therefore, anticoagulant therapy based on direct oral anticoagulants (DOACs) may be needed, despite the bleeding risk. In case of surgery or bleeding, such patients may be concomitantly treated with emicizumab (routine prophylaxis), factor (F)VIII products, and DOAC. Their concomitant presence constitutes a hemostatic challenge. Recent international guidelines stated that data are scarce on the hemostatic balance of plasma samples from patients with HA receiving emicizumab and DOAC.

**Objectives:**

The aim of this observational study was to assess the coagulation of FVIII-deficient plasma spiked with DOAC and emicizumab and to evaluate the effects of FVIII addition.

**Methods:**

Prothrombin time, activated partial thromboplastin time, and thrombin generation (TG) using the calibrated automated thrombogram method were evaluated in aliquots of a commercial severe HA plasma supplemented with emicizumab (0, 12.5, 25, 50, and 100 ng/mL), DOAC (0, 50, 100, 200, and 400 ng/mL of apixaban, rivaroxaban, edoxaban, or dabigatran) and FVIII (0%, 5%, 15%, 50%, and 100%).

**Results:**

DOAC rapidly induced a TG decrease. Emicizumab could counter this effect only for the lowest DOAC dose. FVIII addition to the FVIII-deficient plasma containing a DOAC and emicizumab improved TG and countered the anticoagulant effect of DOAC at ≤100 ng/mL.

**Conclusion:**

Our findings indicate that FVIII can be safely used with emicizumab to counter the anticoagulant effect of DOAC at ≤100 ng/mL. The TG assay is an efficient tool to monitor plasma containing anti-FXa DOAC, but not dabigatran (anti-FIIa).

## Essentials

• Age-related thrombotic events requiring anticoagulation increase in persons with hemophilia A.
• The hemostatic balance with emicizumab, direct oral anticoagulant, and factor (F)VIII is not known.
• FVIII can counter direct oral anticoagulant concentration up to 100 ng/mL in the presence of emicizumab.
• The combined effect of the hemostatic capacity can guide clinicians to manage urgent events.

## 1 Introduction

Hemophilia A (HA) is an inherited bleeding disorder characterized by a quantitative or qualitative coagulation factor (F)VIII defect. For several decades, HA management has been based on intravenous FVIII infusions that have significantly improved life expectancy [[1], [2], [3]]. The prophylaxis regimen (2-3 intravenous infusions of FVIII per week) reduces bleeding events, but is burdensome for patients, thus causing treatment adherence issues [4,5]. Recently, non–factor replacement strategies have emerged, such as the antibody emicizumab that mimics FVIII activity, the small interfering RNA fitusiran that targets antithrombin, and the antibody concizumab against tissue factor (TF) pathway inhibitor [[6], [7], [8]]. Emicizumab is a bispecific monoclonal antibody that ensures the spatial interaction of FIXa with FX, thus mimicking FVIIIa procoagulant activity [9]. The HAVEN studies demonstrated that emicizumab reduces the annual bleeding rate in persons with HA (with or without inhibitors), compared to FVIII products [[10], [11], [12], [13]].

The significant improvement in HA management has led to an increase in quality of life and life expectancy. Consequently, age-related diseases, such as cardiovascular diseases (CVDs), are now observed in persons with HA. It has been shown that persons with HA have a similar or higher risk of hypertension, atherosclerosis, atrial fibrillation, and myocardial infarction compared with the general population [[14], [15], [16], [17]]. Several risk factors of CVD are particularly frequent in persons with HA, for instance, physical inactivity, chronic inflammation, and smoking [18]. CVD management in persons with HA requires fine-tuning of the hemostatic balance to avoid both thrombotic and bleeding events. Indeed, direct oral anticoagulants (DOACs) can be used in persons with HA and CVD, but their effect will counteract that of the prophylaxis treatment [19,20]. Recent international guidelines on the management of CVD in persons with HA recommend antithrombotic treatment for patients with FVIII >20% [21].

Conversely, data on patients on emicizumab prophylaxis are scarce. Yet, the concomitant use of emicizumab and a DOAC in persons with HA leads to new hemostatic challenges. Emicizumab concentration can be assessed using a modified 1-stage assay and an emicizumab calibrator. However, caution is needed to interpret these results because the test is influenced by endogenous FVIII [22]. Emicizumab can interfere with several laboratory tests, such as activated partial thromboplastin time (aPTT) and FVIII dosage, that rely on FVIII and FIX activity. Therefore, they cannot be used to monitor hemostasis in patients on emicizumab-based prophylaxis [23]. Alternative assays, such as chromogenic assays, thrombin generation assays (TGA), and thromboelastography, have been proposed as more reliable methods to monitor coagulation in these patients [[24], [25], [26]]. Decreased thrombin generation (TG) has been associated with the bleeding phenotype severity in persons with HA [27]. Moreover, TGA allowed identification of the synergistic effect of activated prothrombin complex concentrates and emicizumab *in vitro* [28]. TGA is a promising tool for monitoring persons with HA treated with emicizumab because it allows assessing their hemostasis balance regardless of their inhibitor status [29]. Little is known concerning hemostasis in persons with HA receiving both emicizumab and DOAC because routine coagulation tests cannot be used due to the interference of these drugs. Yet, data on their hemostatic balance are needed and are crucial in case of urgent surgery. To this aim, here, we analyzed the effect on TGA of various concentrations of FVIII added to HA plasma sample spiked with emicizumab and different DOAC at different concentrations (within the therapeutic range). This analysis will provide data for the management of persons with HA treated with emicizumab and who require an anticoagulant. These data will also be useful for the management of bleeding events or surgery in persons with HA treated with emicizumab and anticoagulant therapy.

## 2 Methods

### 2.1 Sample preparation

All plasma samples (*N* = 125) were prepared using a commercial FVIII-deficient plasma sample (Cryopep) in which emicizumab (Roche-Chugai), 1 of 4 DOACs (apixaban, rivaroxaban, edoxaban, or dabigatran; Alsachim) and plasma-derived FVIII (Factane, LFB) were added at various doses within the therapeutic range [30]. Specifically, FVIII was added to obtain final concentrations of 0%, 5%, 15%, 50%, and 100% (corresponding to 0, 5, 15, 50, and 100 IU/dL); emicizumab to obtain final concentrations of 0, 12.5, 25, 50, and 100 ng/mL (trough plasma levels between 13.4 and 55.2 ng/mL, depending on the regimen [31]); and DOAC to obtain final concentrations of 0, 50, 100, 200, and 400 ng/mL (50 ng/mL represents a subtherapeutic dose and 400 ng/mL is a supratherapeutic dose). For each drug, a dosage has been performed to ensure that the right amount was added using the corresponding test and reagents on STA R Max analyzer (Stago): emicizumab control/calibrator (r^2^ Diagnostics), ImmunoDef VIII (Stago), and apixaban/rivaroxaban/edoxaban/dabigatran control/calibrator (Stago). For each aliquot, the volume of each added drug represented 1% of the final volume, and therefore, the total added volume did not exceed 3%. The commercial solution of emicizumab has been used. FVIII and DOAC were diluted in sterile water and the control sample was spiked with 3% of sterile water. Supplementary Figure S1 shows the schematic representation of the study design.

### 2.2 Determination of FVIII-deficient plasma characteristics and measurement of prothrombin time and aPTT

Coagulation assays were performed on STA R coagulation analyzer using relevant reagents (Stago). STA-NeoPTimal was used for the measurements of prothrombin time (PT) and some factors (FII, FV, FVII, and FX), STA-PTTA for aPTT (STA-CK-PREST when activated with kaolin) and intrinsic factors, and STA Liquid Fib for fibrinogen using the Clauss method. The relevant deficient plasma references were used for factor measurements (Stago): STA-deficient II, STA-deficient V, STA-deficient VII, STA-deficient X, STA-deficient VIII, STA-deficient IX, STA ImmunoDef XI, STA ImmunoDef XII. STA-ATIII, STACLOT PC, STACLOT PS, and STACLOT APCR were used in functional assays to measure antithrombin, protein C, protein S, and resistance to protein C, respectively.

### 2.3 Measurement of emicizumab and exogenous FVIII activity in plasma

To assess the FVIII-like activity of emicizumab in the FVIII-deficient plasma, a modified 1-stage FVIII assay was used as previously described [32]. This assay was calibrated using the emicizumab calibrator from r^2^ Diagnostics on a STA R Max analyzer. This assay measures the “global” FVIII-like activity due to emicizumab and endogenous and exogenous FVIII. In addition, a chromogenic FVIII assay and bovine reagents were used to determine the exogenous FVIII activity.

### 2.4 TGA

TGA was used to assess the coagulation of plasma samples supplemented with emicizumab, DOAC, and exogenous FVIII. TGA was performed in duplicate using the calibrated automated thrombogram method [33], a fluorometer (Fluoroskan Ascent, ThermoLab Systems) equipped with a dispenser, PPP reagent (5 pM TF, 4 μM phospholipids, final concentrations), PPP_low_ reagent (1 pM TF, 4 μM phospholipids, final concentrations), and FluCa kit (Stago). PPP reagent triggers TG with an intermediate TF concentration while PPP_low_ reagent uses a low TF concentration. Before each assay, plates were incubated at 37 °C for 10 minutes. Raw data were analyzed using the Thrombinoscope software (Stago). Endogenous thrombin potential (ETP) and thrombin peak were the main parameters analyzed. ETP is the area under the TG curve, reflecting the total amount of thrombin generated throughout the assay. Thrombin peak represents the maximum thrombin concentration reached during the coagulation process. As this was an observational study, no statistical analysis was performed.

## 3 Results

### 3.1 Characteristics of the FVIII-deficient plasma

The Table summarizes the result of coagulation parameter analysis in the FVIII-deficient plasma. Analysis of the FVIII activity before spiking confirmed that the purchased plasma had an FVIII concentration of <1% (ie, severe HA) and that all the other coagulation factors were normal or just below the normal levels. In this FVIII-deficient plasma, aPTT (97.1 seconds) and aPTT with kaolin (86.7 seconds) were prolonged, whereas PT (14.4 seconds) and fibrinogen concentration (3.3 g/L) were within the reference ranges. ETP and thrombin peak were below the reference range, as expected.

**Table:** Factor VIII–deficient plasma characteristics.

Coagulation parameters					
PT (s)	14.4 [<15.2]	Factor XII (%)	76 [80-120]	AT (%)	93 [80-120]
INR	1.09 [<1.2]	Factor XI (%)	87 [80-120]	Protein C (%)	123 [80-120]
aPTT (s)	97.1 [<37]	Factor IX (%)	96 [80-120]	Protein S (%)	85 [80-120]
aPTT kaolin (s)	86.7 [<34]	Factor VIII (%)	<1 [80-120]	APCR (s)	207 [>120]
Fibrinogen (g/L)	3.27 [2-4]	Factor VII (%)	93 [80-120]		
		Factor X (%)	78 [80-120]		
		Factor V (%)	67 [80-120]		
		Factor II (%)	91 [80-120]		
**Thrombin generation assay**					
PPP reagent (Intermediate TF)		PPP_low_ reagent (Low TF)			
ETP (nM·min)	724 [1226-1487]	ETP (nM·min)	203 [837-1196]		
Peak (nM)	56 [209-276]	Peak (nM)	9 [82-144]		
Lag time (min)	2.0 [3.0-3.8]	Lag time (min)	3.0 [4.7-6.79]		
ttPeak (min)	6.5 [5.8-7.3]	ttPeak (min)	12.8 [9.74-11.98]		

Reference ranges are provided in brackets.

APCR, activated protein C resistance; aPTT, activated partial thromboplastin time; AT, antithrombin; ETP, endogenous thrombin potential; INR, International Normalized Ratio; PT, prothrombin time; ttPeak, time to peak.

### 3.2 DOAC effect on FVIII-deficient plasma containing emicizumab

#### 3.2.1 Coagulation parameters

In the presence of emicizumab (0-100 ng/mL), PT was not affected upon addition of apixaban or dabigatran (range, 14.9-19.2 seconds), but was prolonged after addition of rivaroxaban or edoxaban (range, 14.3-29.9 seconds). Anti-FXa DOAC increased aPTT in a dose-dependent manner (range, 74.3-116.9 seconds at 50 ng/mL to 119-169.2 seconds at 400 ng/mL); aPTT reached 180 seconds with 50 ng/mL of dabigatran. In presence of emicizumab, aPTT was shortened, whatever the dose or DOAC used (Supplementary Table S1).

#### 3.2.2 TG

**Figure 1:**
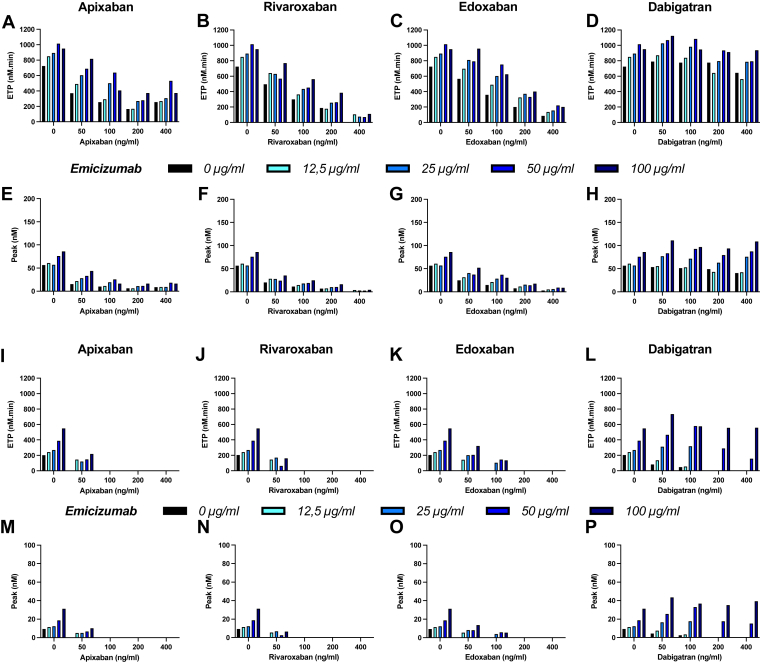
Direct oral anticoagulant effect on thrombin generation assay of a factor (F)VIII–deficient plasma spiked with emicizumab. (A–D and I–L) Endogenous thrombin potential (ETP) and (E–H and M–P) thrombin peak obtained with thrombin generation assay using the (A–H) intermediate tissue factor and (I–P) low tissue factor concentrations in a FVIII-deficient (FVIII, <1%) plasma sample complemented with the indicated doses of emicizumab and after addition of the indicated direct oral anticoagulant (0, 50, 100, 200, and 400 ng/mL).

In FVIII-deficient plasma spiked with increasing emicizumab concentrations, ETP ranged between 725 and 1025 nM·min, and the thrombin peak from 56 to 86 nM when TG was initiated with an intermediate TF concentration. Similarly, ETP was between 203 and 550 nM·min and thrombin peak between 9 and 31 nM when TG was triggered with a low TF concentration (Figure 1). Interassay coefficient of variation (CV) is 10.65% for ETP and 12.50% for peak thrombin while intra-assay CV ranges from 0.28% to 13.82% for ETP and from 0.32% to 11.61% for thrombin peak.

Apixaban addition decreased TG in a dose-dependent manner in plasma with/without emicizumab (Figure 1). ETP (TGA with intermediate TF) ranged from 372 to 817 nM·min, 256 to 638 nM·min, 166 to 673 nM·min, and 257 to 531 nM·min after addition of 50, 100, 200 and 400 nM·min apixaban, respectively (Figure 1A). Thrombin peak ranged from 15 to 44 nM, 10 to 26 nM, 6 to 16 nM, and 9 to 18 nM after addition of 50, 100, 200, and 400 ng/mL apixaban, respectively (Figure 1E). This apixaban-induced effect was only slightly compensated by emicizumab. TG triggered with low TF could be measured only at the lowest dose of apixaban (50 ng/mL) and in samples with emicizumab. ETP ranged from 0 to 218 nM·min and thrombin peak from 0 to 10 nM (Figure 1I, M).

Rivaroxaban addition gave similar results. In the TGA with intermediate TF, ETP ranged from 496 to 770 nM·min, 300 to 563 nM·min, 180 to 387 nM·min, and 0 to 111 nM·min for 50, 100, 200, and 400 nM·min rivaroxaban, respectively (Figure 1B). Thrombin peak ranged from 20 to 35 nM, 11 to 25 nM, 7 to 16 nM, and 0 to 4 nM for 50, 100, 200, and 400 ng/mL, respectively (Figure 1F). TG triggered with intermediate TF concentration gave ETP comprised between 0 and 171 nM·min and thrombin peak between 0 and 7 nM (Figure 1J, N) with 50 ng/mL rivaroxaban.

Similarly, in the TGA with intermediate TF concentration, ETP ranged from 567 to 957 nM·min, 360 to 753 nM·min, 202 to 402 nM·min, and 86 to 222 nM·min for 50, 100, 200, and 400 nM·min edoxaban, respectively (Figure 1C). Thrombin peak ranged from 25 to 52 nM, 15 to 37 nM, 8 to 18 nM, and 3 to 9 nM for 50, 100, 200, and 400 ng/mL, respectively (Figure 1G). Unlike apixaban and rivaroxaban, TG triggered with low TF concentration could be measured also in the presence of 100 ng/mL edoxaban. ETP ranged from 0 to 322 nM·min and 0 to 145 nM·min for 50 and 100 ng/mL edoxaban. Thrombin peak ranged from 0 to 7 nM and from 0 to 6 nM at 50 ng/mL and 100 ng/mL edoxaban, respectively (Figure 1K, O).

On the other hand, dabigatran supplementation only slightly affected TG in the TGA with intermediate TF concentration. ETP ranged from 792 to 1125 nM·min, 777 to 1084 nM·min, 643 to 936 nM·min, and 644 to 937 nM·min with 50, 100, 200, and 400 nM·min dabigatran, respectively (Figure 1D). Thrombin peak ranged from 53 to 111 nM, 51 to 97 nM, 43 to 94 nM, and 41 to 109 nM in the presence of 50, 100, 200, and 400 ng/mL dabigatran, respectively (Figure 1H). Unlike the other tested DOAC (anti-FXa molecules), TG triggered with low TF concentration could be measured at all dabigatran (anti-FIIa DOAC) doses. ETP ranged from 81 to 735 nM·min, 47 to 581 nM·min, 0 to 556 nM·min, and 0 to 557 nM·min for 50, 100, 200 and 400 ng/mL dabigatran, respectively. Thrombin peak ranged from 4 to 43 nM, 3 to 37 nM, 0 to 35 nM, and 0 to 39 nM at 50, 100, 200, and 400 ng/mL dabigatran, respectively (Figure 1L, P).

TG seemed to reach a plateau at the therapeutic dose of emicizumab (50 ng/mL). Increasing the DOAC dose decreased ETP and thrombin peak; however, emicizumab (at all doses) still exerted its procoagulant activity.

### 3.3 Effect of FVIII supplementation on FVIII-deficient plasma containing emicizumab and DOAC

#### 3.3.1 Coagulation parameters

Upon FVIII addition (different doses) to FVIII-deficient plasma spiked with emicizumab and DOAC, PT was not affected whatever the dose used (ranging from 14.3 to 18.5 seconds for all ranges of emicizumab and apixaban used). The aPTT values were not significantly altered by FVIII in the presence of emicizumab, ranging, eg, from 25.1 to 33.3 seconds for all emicizumab (except 0 ng/mL) and apixaban doses (Supplementary Table S2).

#### 3.3.2 TG

**Figure 2:**
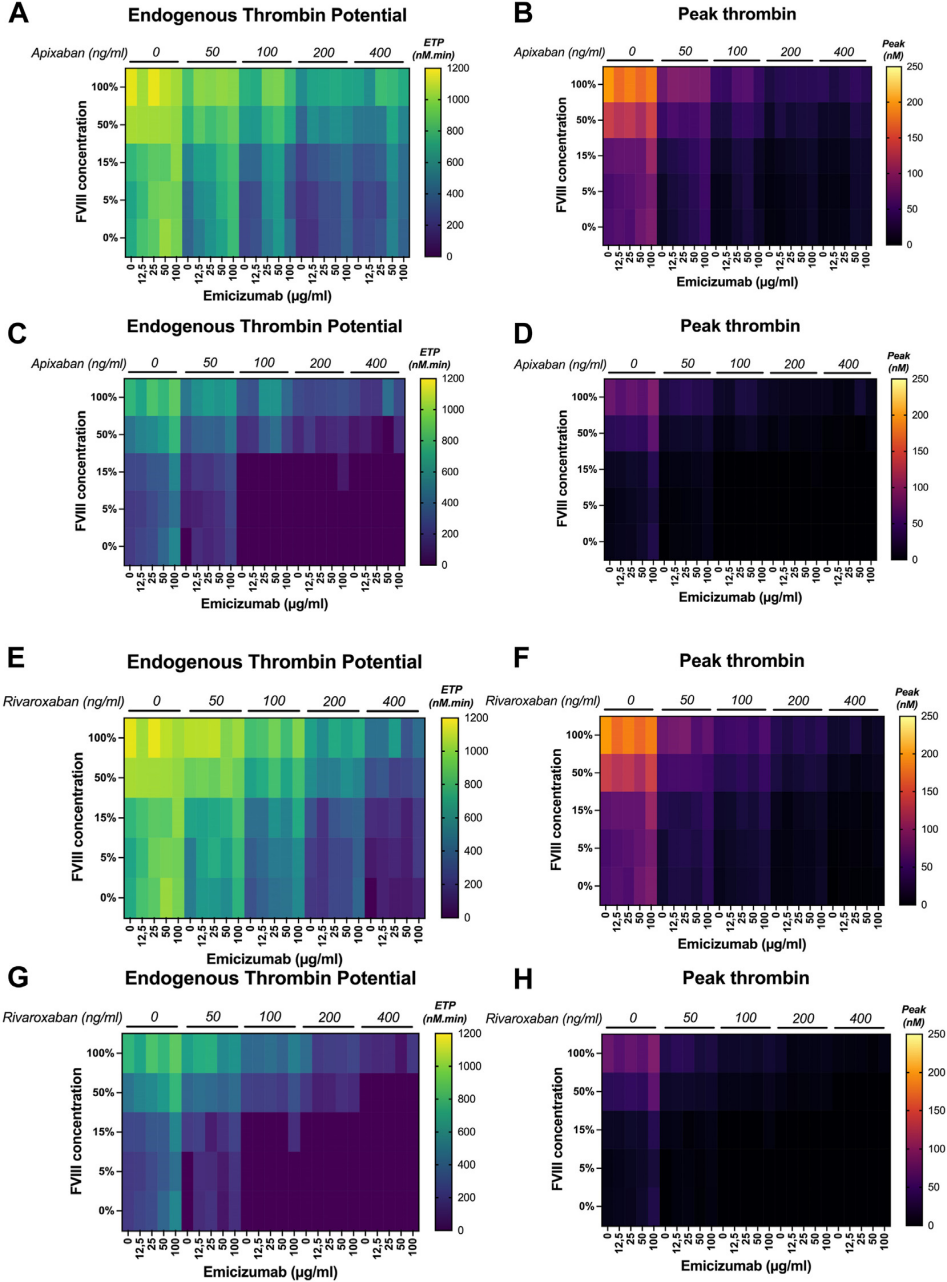
Thrombin generation in a factor (F)VIII–deficient plasma containing emicizumab, apixaban or rivaroxaban, and FVIII. The heatmaps show endogenous thrombin potential (ETP) and thrombin peak in the presence of (A–D) apixaban and (E–H) rivaroxaban. Thrombin generation assay was carried out with (A, B, E, F) intermediate tissue factor and (C, D, G, H) low tissue factor (concentrations. The doses of emicizumab (*x* axes) and of each direct oral anticoagulant (top of each heatmap) in each aliquot are indicated. As such, for a single direct oral anticoagulant dose (ie, 0 ng/mL), 5 horizontal rectangles correspond to each emicizumab concentration (0-100 μg/mL from left to right) and each row represents an FVIII concentration. A graphical version of these results is available in Supplementary Figures S3 (apixaban) and S4 (rivaroxaban).

In the absence of FVIII, ETP (TGA with intermediate TF concentration) increased from 372 to 817, 256 to 408, 166 to 373, and 256 to 531 nM·min and thrombin peak from 15 to 44, 10 to 26, 6 to 16, and 9 to 18 nM in the presence of emicizumab and 50, 100, 200, and 400 ng/mL apixaban, respectively (Figure 2A, B). Supplementation with 15% FVIII increased ETP from 523 to 844, 393 to 668, 225 to 402, and 307 to 581 nM·min and thrombin peak from 25 to 49, 16 to 28, 9 to 18, and 10 to 20 nM in plasma samples with 50, 100, 200, and 400 ng/mL apixaban, respectively. As observed for emicizumab, high FVIII doses led to minor differences. Indeed, increasing the FVIII dose (50%-100%) in plasma containing emicizumab increased ETP from 769 to 1002, 642 to 961, 382 to 730, and 466 to 823 nM·min and thrombin peak from 45 to 90, 27 to 62, 16 to 36, and 16 to 40 nM in plasma spiked with 50, 100, 200, and 400 ng/mL apixaban, respectively. Data on the time-to-peak parameter are accessible in Supplementary Figure S2. When TGA was carried out with low TF concentration, TG could not be measured in samples spiked with the lowest FVIII doses (5%-15%) but gave profiles similar to those obtained with intermediate TF concentration for the highest FVIII dose (Figure 2C, D). In samples with 200 ng/mL apixaban, ETP ranged from 0 to 75 nM·min with 15% FVIII and from 109 to 290 nM·min with 50% to 100% FVIII. Thrombin peak followed the same trend: 0 to 3 nM with 15% FVIII and 5 to 15 nM with 50% to 100% FVIII.

In the absence of FVIII, ETP (TGA with intermediate TF concentration) varied from 496 to 770, 300 to 563, 180 to 387, and 0 to 111 nM·min and thrombin peak from 20 to 35, 11 to 25, 7 to 16, and 0 to 4 nM in plasma with emicizumab and 50, 100, 200, and 400 ng/mL rivaroxaban, respectively (Figure 2E, F). Supplementation with 15% FVIII increased ETP from 695 to 865, 430 to 727, 174 to 374, and 142 to 205 nM·min and thrombin peak from 33 to 42, 18 to 32, 7 to 16, and 6 to 8 nM in plasma with emicizumab and 50, 100, 200, and 400 ng/mL rivaroxaban, respectively. Similar to emicizumab, spiking with high FVIII doses only slightly changed the results. Specifically, increasing the FVIII dose (50%-100%) increased ETP from 921 to 1071, 685 to 971, 490 to 745, and 227 to 655 nM·min and thrombin peak from 51 to 84, 33 to 57, 20 to 36, and 9 to 29 nM in plasma with emicizumab and 50, 100, 200, and 400 ng/mL rivaroxaban, respectively. When TGA was carried out with low TF concentration, TG could not be measured after spiking with the lowest FVIII doses (5%-15%) but showed a profile similar to that with intermediate TF concentration for the highest FVIII doses used (Figure 2G, H). Indeed, in plasma with emicizumab and 200 ng/mL rivaroxaban, the anticoagulation activity was too strong to generate enough thrombin in the presence of only 15% FVIII; conversely, ETP ranged from 100 to 415 nM·min with 50% to 100% FVIII. Thrombin peak followed the same trend and ranged between 4 and 18 nM with 50% to 100% FVIII.

**Figure 3:**
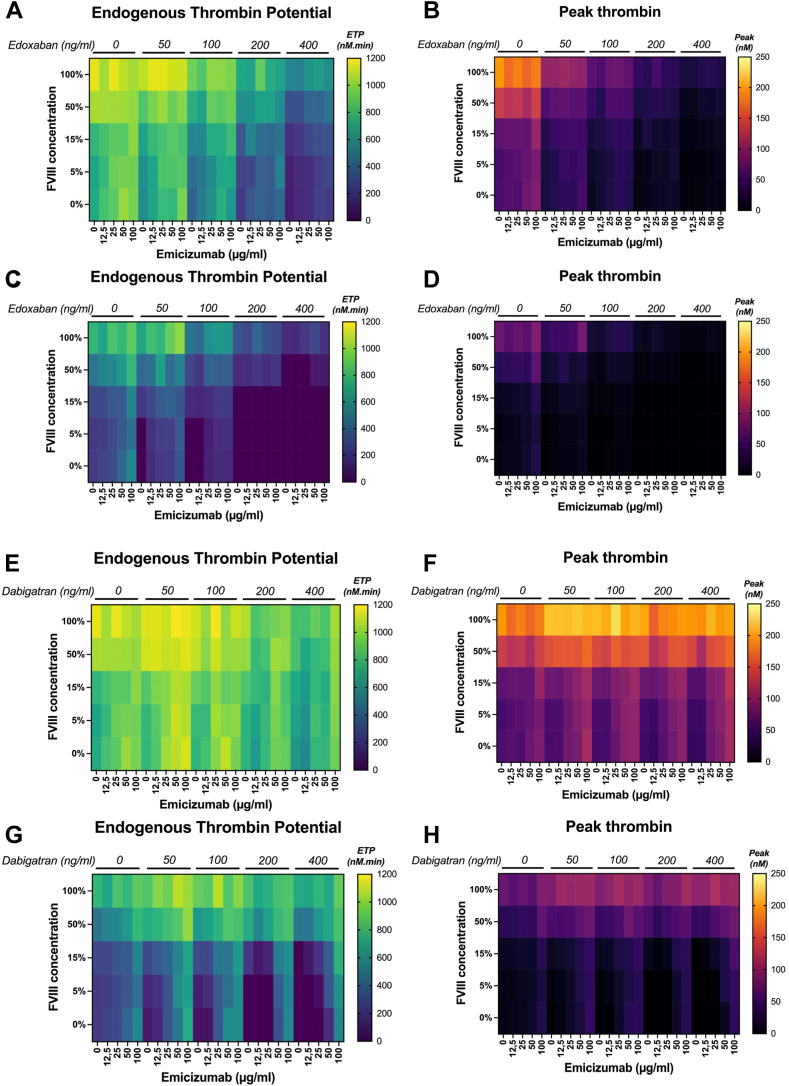
Thrombin generation in a factor (F)VIII–deficient plasma containing emicizumab, edoxaban or dabigatran, and FVIII. The heatmaps show endogenous thrombin potential (ETP) and thrombin peak in the presence of (A–D) edoxaban and (E–H) dabigatran. Thrombin generation assay was carried out with (A, B, E, F) intermediate tissue factor and (C, D, G, H) low tissue factor concentrations. The doses of emicizumab (*x* axis) and of each direct oral anticoagulant (top of each heatmap) in each aliquot are indicated. As such, for a single direct oral anticoagulant dose (ie, 0 ng/mL), 5 horizontal rectangles correspond to each emicizumab concentration (0-100 μg/mL from left to right) and each row represents an FVIII concentration. A graphical version of these results is available in Supplementary Figures S5 (edoxaban) and S6 (dabigatran).

In the absence of FVIII, ETP (TGA with intermediate TF dose) ranged from 567 to 957, 360 to 753, 202 to 402, and 86 to 222 nM·min and thrombin peak from 25 to 52, 15 to 37, 8 to 18, and 3 to 9 nM in plasma with emicizumab and 50, 100, 200, and 400 ng/mL edoxaban, respectively (Figure 3A, B). Spiking with 15% FVIII increased the ETP from 688 to 889, 547 to 820, 325 to 455, and 160 to 310 nM·min and thrombin peak from 34 to 50, 25 to 41, 14 to 27, and 6 to 14 nM in plasma with emicizumab and 50, 100, 200 and 400 ng/mL edoxaban, respectively. As observed with emicizumab alone, high FVIII doses did not strongly affect the TGA results. Upon spiking with 50% to 100% FVIII, ETP ranged from 867 to 1152, 749 to 995, 577 to 915, and 291 to 661 nM·min, and thrombin peak from 56 to 105, 34 to 74, 27 to 55, and 13 to 36 nM in plasma with emicizumab and 50, 100, 200, and 400 ng/mL edoxaban, respectively. In TGA with low TF concentration, TG was undetectable upon addition of 5% to 15% FVIII but showed a profile similar to that with intermediate TF concentration for the highest FVIII doses (Figure 3C, D). In plasma with emicizumab and 200 ng/mL edoxaban, the anticoagulant activity was too strong to generate enough thrombin in the presence of 15% FVIII, whereas ETP ranged from 143 to 387 nM·min with 50% to 100% FVIII. Thrombin peak followed the same trend and ranged between 6 and 18 nM with 50% to 100% FVIII.

In the absence of FVIII, ETP (TGA with intermediate TF concentration) ranged from 792 to 1125, 777 to 1084, 643 to 936, and 644 to 937 nM·min and thrombin peak from 53 to 111, 51 to 97, 43 to 94, and 41 to 109 nM in plasma with emicizumab and 50, 100, 200, and 400 ng/mL dabigatran, respectively (Figure 3E, F). Spiking with 15% FVIII increased ETP from 875 to 1060, 878 to 999, 804 to 978, and 717 to 984 nM·min and thrombin peak from 75 to 110, 72 to 105, 71 to 101, and 60 to 121 nM in plasma with emicizumab and 50, 100, 200, and 400 ng/mL dabigatran, respectively. Similar to emicizumab, high FVIII concentrations did not strongly affect the TGA results. Indeed, increasing the FVIII dose (50%-100%) increased ETP from 867 to 1152, 749 to 995, 577 to 915, and 291 to 661 nM·min and thrombin peak from 56 to 105, 34 to 74, 27 to 55, and 13 to 36 nM in plasma with emicizumab and 50, 100, 200, and 400 ng/mL dabigatran, respectively. When TGA was carried out with low TF concentration, TG was undetectable after spiking with 5% to 15% FVIII, but showed a profile similar to TGA with intermediate TF after spiking with the highest FVIII doses (Figure 3G, H). For instance, in the plasma with emicizumab and 200 ng/mL dabigatran, the anticoagulant activity was too strong to generate enough thrombin with 15% FVIII, but ETP and thrombin peak ranged from 143 to 387 nM·min and from 6 to 18 nM with 50% to 100% FVIII.

TGA allowed monitoring coagulation in plasma-containing drugs that exert contradictory effects (DOAC, emicizumab, and FVIII) and showed the procoagulant activity of emicizumab, even in the presence of DOAC. FVIII supplementation had a slight additive effect when used at low dose in combination with emicizumab.

## 4 Discussion

Our study gives an overview of the hemostatic capacity of FVIII-deficient plasma spiked or not with different concentrations of emicizumab, DOAC, and FVIII. Our results could help clinicians to choose the FVIII dose to be used before urgent surgery or during a bleeding event for patients on emicizumab prophylaxis and taking or not a DOAC for a CVD. Our findings support the use of FVIII products, whatever the emicizumab dose, because emicizumab alone does not allow a sufficient TG level when a DOAC is concomitantly present in the plasma. Indeed, 100% FVIII completely masked emicizumab effect, as already documented, and did not fully compensate for the anticoagulant effect of a DOAC at a concentration >100 ng/mL.

We first assessed coagulation using routine tests and observed an increase in PT with DOAC supplementation and also a superior effect of rivaroxaban compared with apixaban, in agreement with the literature [34]. As expected, aPTT was greatly shortened after spiking with emicizumab. In persons with HA on emicizumab-based prophylaxis and treated with a DOAC, PT, and aPTT should not be used to monitor their coagulation. FVIII had a bigger effect on TGA than emicizumab, certainly due to its higher affinity for its substrates [9]. Several studies analyzed supplementation of FVIII or recombinant FVIIa in persons with HA undergoing surgery and concluded that hemostatic supplementation is crucial for major surgical interventions, while it could be skipped for some minor surgeries [35]. Some studies assessed TGA to manage bypassing agents (activated prothrombin complex concentrates or recombinant FVIIa) or FVIII product dose adjustment in a few persons with HA (with or without inhibitors) on emicizumab-based prophylaxis. They found that TGA could be used to efficiently monitor the hemostatic balance of plasma samples containing emicizumab and procoagulant products and allowed the efficient and safe surgical management of such patients [29,36]. Dargaud et al. [29] showed a successful adjustment of the bypassing agent dosage using TGA in 1 person with HA and inhibitors on emicizumab and undergoing surgery. They also used TGA to manage arterial bleeding by bypassing agent infusions in the same patient [29]. Our study is in line with the literature and is in favor of FVIII supplementation for patients undergoing surgery to avoid any bleeding event or to treat a bleeding event. FVIII supplementation appears to be safe because it only has a weak additional effect with emicizumab at the lowest doses used and allows approaching the normal TG range.

Spiking with DOAC produced different effects depending on the factor they target. Anti-FXa DOAC decreased ETP and peak thrombin from 50 ng/mL, but not dabigatran (anti-FIIa DOAC). Despite their similar anticoagulant profile by TGA, DOAC targeting FXa showed some discrepancies. Indeed, at 400 ng/mL, apixaban did not further decrease TG compared with 200 ng/mL, unlike rivaroxaban and edoxaban. Emicizumab partially reversed the anticoagulant effect of anti-FXa DOAC, as indicated by the continuous decrease of ETP and thrombin peak, reaching low values at the highest doses. Nonetheless, these data suggest that DOAC concentration should be monitored before surgery because doses >100 ng/mL produce a strong anticoagulant effect. In this case, FVIII should be supplemented at high dose, over the emicizumab prophylaxis, to evaluate the DOAC effect and a complementary approach may be considered. Below 100 ng/mL of DOAC, FVIII seems sufficient to counter its procoagulant effect. While in persons without hemophilia, a surgery performed with a dose of DOAC <50 ng/mL or <100 ng/mL does not result in significant blood loss, this result cannot be extrapolated to persons with HA [37,38]. However, it is interesting to determine a threshold for FVIII supplementation depending on the DOAC, its dosage and the emicizumab dosage in persons with HA to help clinicians to manage urgent cases.

As routine coagulation tests cannot be used to monitor complex situations (i.e. plasma samples containing emicizumab), TGA appears as a solid assay to evaluate hemostasis in such samples [22,39]. In DOAC-free plasma containing FVIII, the ETP values obtained with low TF TGA were similar to those reported by previous studies performed with plasma from persons with mild, moderate, and severe HA [40,41], but were lower than in other studies [42,43]. TGA is not a standardized test and many different protocols can be found in the literature. We chose to perform the test with 2 TF concentrations to cover the maximum range of TG due to the complexity of the tested plasma samples. TGA has been used to monitor the hemostatic effect of molecules that mimic FVIII activity (emicizumab and more recently mim8) with promising results [44,45]. TGA can be triggered by various components (eg, ellagic acid, TF, FXIa, and FIXa), thus giving different TG profiles. Among these components, FXIa is interesting for carrying out TGA of plasma samples that contain bispecific antibodies mimicking FVIII activity (eg, emicizumab). FXIa supplementation allows producing a large amount of FIXa that no longer constitutes a limiting factor for TG [46]. In our study, in the absence of DOAC, a normal ETP value was not observed even with 100% FVIII. This could be explained by the limited FIXa available for emicizumab. It could be interesting to use TGA with FXIa in the same conditions. Indeed, in TGA, FXIa generates more thrombin and accelerates TG onset compared with TF [39]. Lund et al. tested the effects of the combination of FVIII and mim8 on TG triggered by FXIa and found an additive effect of the 2 drugs when FVIII was used at low dose, which is similar to our observations with emicizumab [47]. In line with the literature, our data also indicate that TGA allows monitoring plasma samples containing anti-FXa DOAC, but not dabigatran (anti-FIIa DOAC) [48]. Although TG decreased with increasing doses of dabigatran, its effect on ETP and thrombin peak was rather limited and did not reflect the anticoagulant effect of this DOAC. Time to peak and to a lesser extent, lag time were the parameters most influenced by dabigatran (data not shown) and should be monitored in persons with HA on dabigatran [49]. However, we choose to not display them because emicizumab displays a delay kinetic response on TG traduced by a lengthening in time to peak and lag time while exerting a procoagulant effect.

Recent recommendations on DOAC treated persons with HA, undergoing cardiac surgery, suggest FVIII peak levels of 80% to 100% [21]. Our data are in line with these recommendations and support FVIII supplementation, whether emicizumab is present or not. Furthermore, in persons with HA who take emicizumab and DOAC and who require urgent, unplanned surgery, FVIII supplementation should be ≥100%, in function of the DOAC dose. As already known, special care should be taken with rivaroxaban because it seems to exert a stronger anticoagulant effect at high doses. Besides HA, emicizumab has been repositioned for von Willebrand disease (VWD) management and showed promising results for reducing bleeding events in patients with type 3 VWD, with/without alloantibodies. It also increases thrombus formation *in vitro* in patients with type 2N VWD [[50], [51], [52]]. Therefore, it would be interesting to test whether its beneficial effect on TG is still present in patients with VWD receiving a DOAC.

The main limitation of the study is the evaluation of only one FVIII-deficient plasma sample (severe HA), which is not representative of the clinical routine, but the use of this plasma allows evaluation of a large panel of conditions. Moreover, it could be interesting to perform the same study using plasma samples from persons with mild and moderate HA. In our study, the highest FVIII dose used (100%) had a limited effect in FVIII-deficient plasma spiked with high doses of DOAC. It is important to determine whether 150% FVIII would further increase TG and to what extent. Repeating this study at other centers could be interesting due to the inherent TGA variability.

To conclude, our study brought insights into the effect on TG of FVIII supplementation in a FVIII-deficient plasma spiked with emicizumab and with different DOAC (increasing concentrations). Our findings demonstrated the importance of FVIII supplementation in the presence of emicizumab to counter the anticoagulant effect induced by DOAC. FVIII and emicizumab can be safely used together because they do not potentiate each other. Our results bring data on the hemostatic response of complex plasma samples using TGA; however, more studies are needed to evaluate *in vivo* combinations.

## Supplementary material

Supplementary Figure S1 **Schematic representation of the study design.** For a single dose of emicizumab, every DOAC at every dose have been spiked as well as every concentration of FVIII. Thrombin generation assays were done with intermediate (PPP reagent) and low (PPP_low_ reagent) tissue factor concentrations. Supplementary Figure S2. **DOAC effect on ttPeak parameter on TGA in a FVIII-deficient plasma spiked with emicizumab.** The heatmaps show the time to peak in the presence of apixaban (**A, B**), rivaroxaban (**C, D**), edoxaban (**E, F**) and dabigatran (**G, H**). TGA was carried out with intermediate TF (**A, C, E, G**) and low TF concentrations (**B, D, F, H**). The doses of emicizumab (x axes) and of each DOAC (top of each heatmap) in each aliquot are indicated. As such, for a single DOAC dose (ie 0 ng/ml), 5 horizontal rectangles correspond to each emicizumab concentration (0 to 100 μg/ml from left to right) and each row represents an FVIII concentration. Dark squares represent conditions not producing enough thrombin to measure ttPeak. A different scale has been used for dabigatran measured with PPPlow due to higher values (**H**). Supplementary Figure S3. **Apixaban effect on TGA in a FVIII-deficient plasma spiked with emicizumab.** ETP and thrombin peak measurement by TGA in a FVIII-deficient (FVIII <1%) plasma sample after addition, or not, of emicizumab and apixaban at the indicated doses. TGA was triggered with intermediate TF (**A-J**) and low TF concentrations (**K-T**). Supplementary Figure S4. **Rivaroxaban effect on TGA in a FVIII-deficient plasma spiked with emicizumab.** ETP and thrombin peak measurement by TGA in a FVIII-deficient (FVIII <1%) plasma sample after addition, or not, of emicizumab and rivaroxaban at the indicated doses. TGA was triggered with intermediate TF (**A-J**) and low TF concentrations (**K-T**). Supplementary Figure S5. **Edoxaban effect on TGA in a FVIII-deficient plasma spiked with emicizumab.** ETP and thrombin peak measurement by TGA in a FVIII-deficient (FVIII <1%) plasma sample after addition, or not, of emicizumab and edoxaban at the indicated doses. TGA was triggered with intermediate TF (**A-J**) and low TF concentrations (**K-T**). Supplementary Figure S6. **Dabigatran effect on TGA in a FVIII-deficient plasma spiked with emicizumab.** ETP and thrombin peak measurement by TGA in a FVIII-deficient (FVIII <1%) plasma sample after addition, or not, of emicizumab and dabigatran at the indicated doses. TGA was triggered with intermediate TF (**A-J**) and low TF concentrations (**K-T**).
