# Supplementary material for: Thrombin generation to evaluate the complex hemostatic balance of hemophilia A plasma containing direct oral anticoagulant and supplemented by factor VIII
Source: Res Pract Thromb Haemost. 2024 Sep 23;8(7):102576. doi: 10.1016/j.rpth.2024.102576 (PMC11532490; doi:10.1016/j.rpth.2024.102576)
Supplement: Supplementary Figure S1 — Schematic representation of the study design. For a single dose of emicizumab, every DOAC at every dose have been spiked as well as every concentration of FVIII. Thrombin generation assays were done with intermediate (PPP reagent) and low (PPPlow reagent) tissue factor concentrations. Supplementary Figure S2. DOAC effect on ttPeak parameter on TGA in a FVIII-deficient plasma spiked with emicizumab. The heatmaps show the time to peak in the presence of apixaban (A, B), rivaroxaban (C, D), edoxaban (E, F) and dabigatran (G, H). TGA was carried out with intermediate TF (A, C, E, G) and low TF concentrations (B, D, F, H). The doses of emicizumab (x axes) and of each DOAC (top of each heatmap) in each aliquot are indicated. As such, for a single DOAC dose (ie 0 ng/ml), 5 horizontal rectangles correspond to each emicizumab concentration (0 to 100 μg/ml from left to right) and each row represents an FVIII concentration. Dark squares represent conditions not producing enough thrombin to measure ttPeak. A different scale has been used for dabigatran measured with PPPlow due to higher values (H). Supplementary Figure S3. Apixaban effect on TGA in a FVIII-deficient plasma spiked with emicizumab. ETP and thrombin peak measurement by TGA in a FVIII-deficient (FVIII <1%) plasma sample after addition, or not, of emicizumab and apixaban at the indicated doses. TGA was triggered with intermediate TF (A-J) and low TF concentrations (K-T). Supplementary Figure S4. Rivaroxaban effect on TGA in a FVIII-deficient plasma spiked with emicizumab. ETP and thrombin peak measurement by TGA in a FVIII-deficient (FVIII <1%) plasma sample after addition, or not, of emicizumab and rivaroxaban at the indicated doses. TGA was triggered with intermediate TF (A-J) and low TF concentrations (K-T). Supplementary Figure S5. Edoxaban effect on TGA in a FVIII-deficient plasma spiked with emicizumab. ETP and thrombin peak measurement by TGA in a FVIII-deficient (FVIII <1%) plasma sample after addition, or n [file mmc1.pptx]

## Slide 1
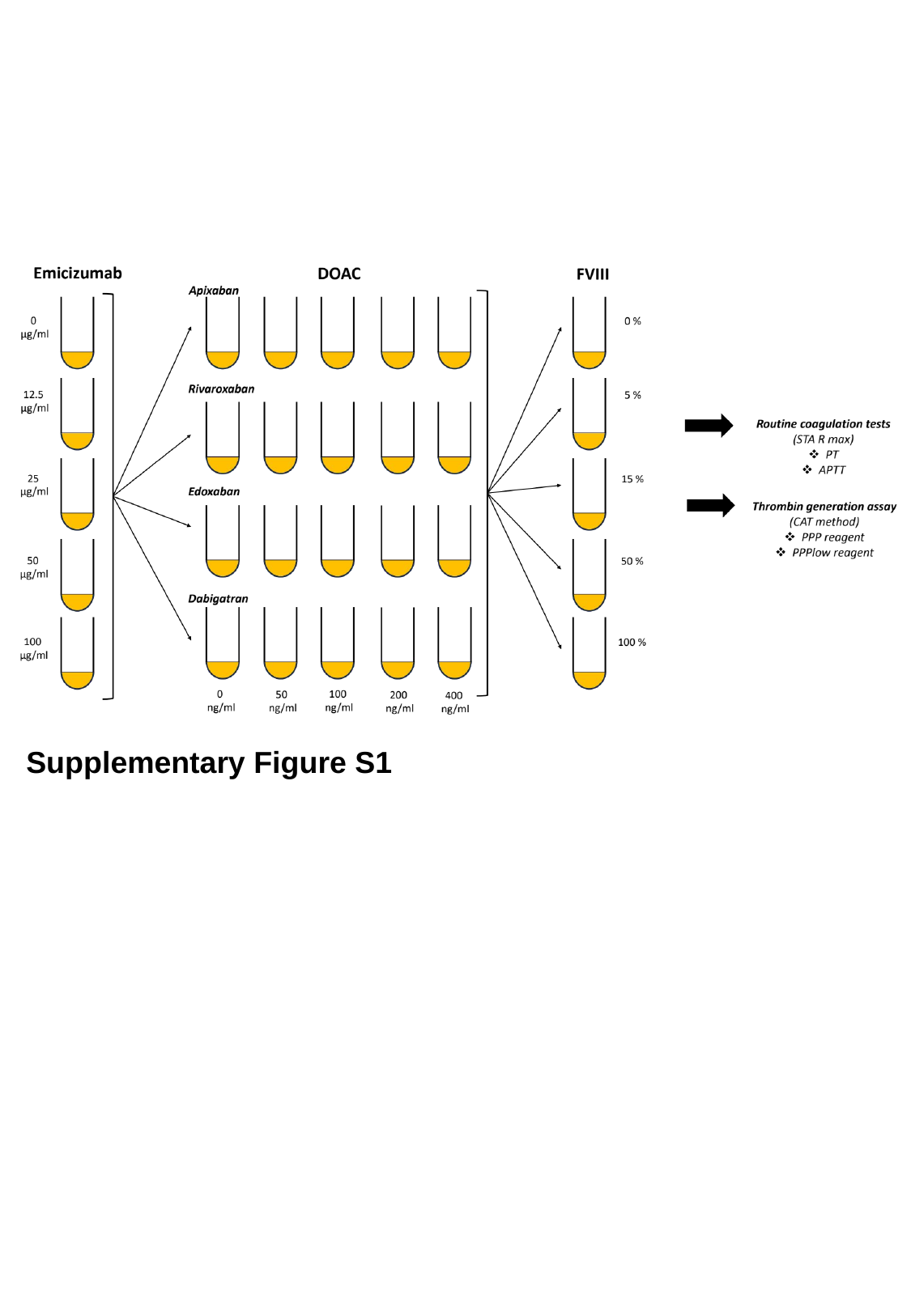

Supplementary Figure S1

## Slide 2
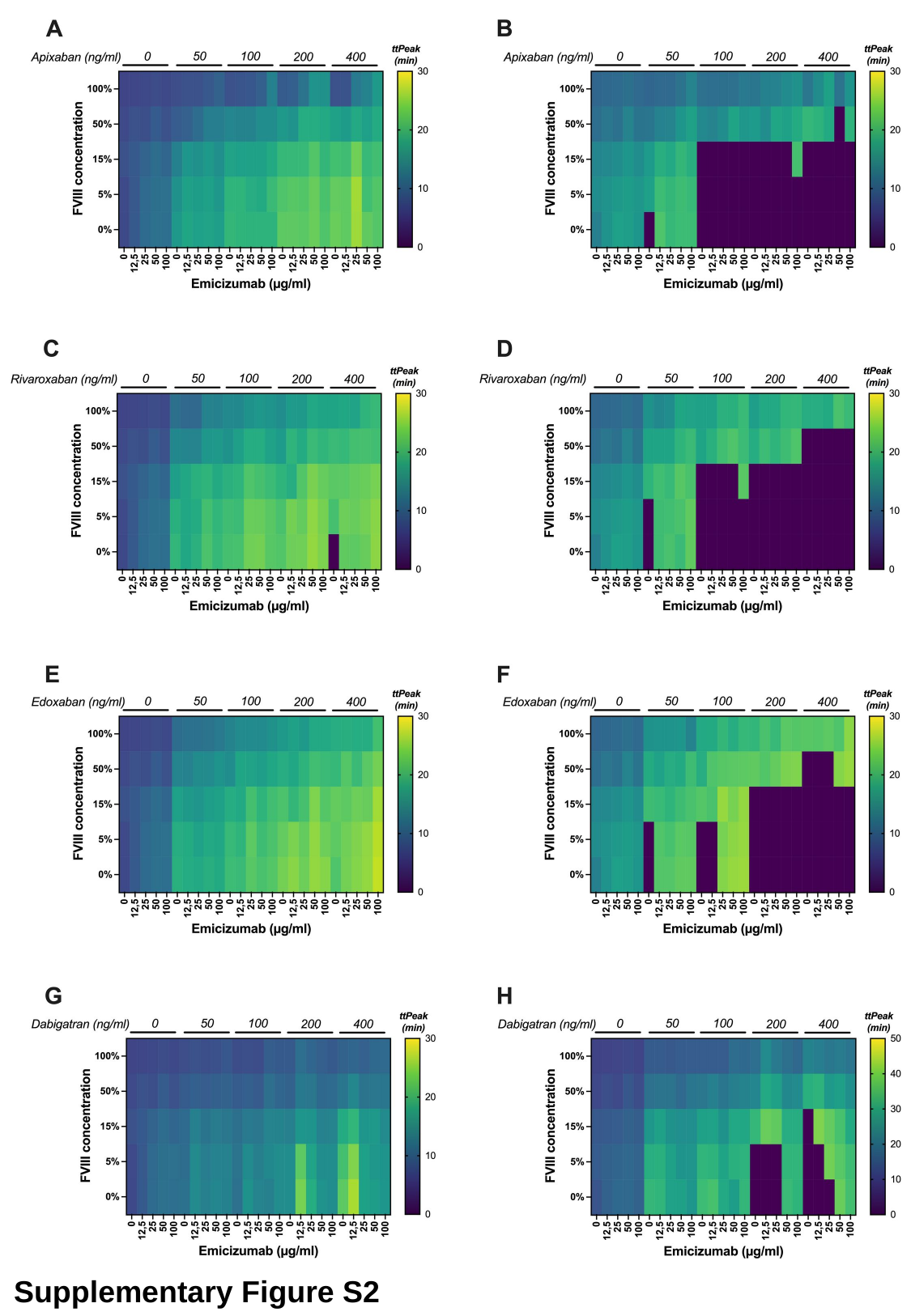

Supplementary Figure S2

## Slide 3
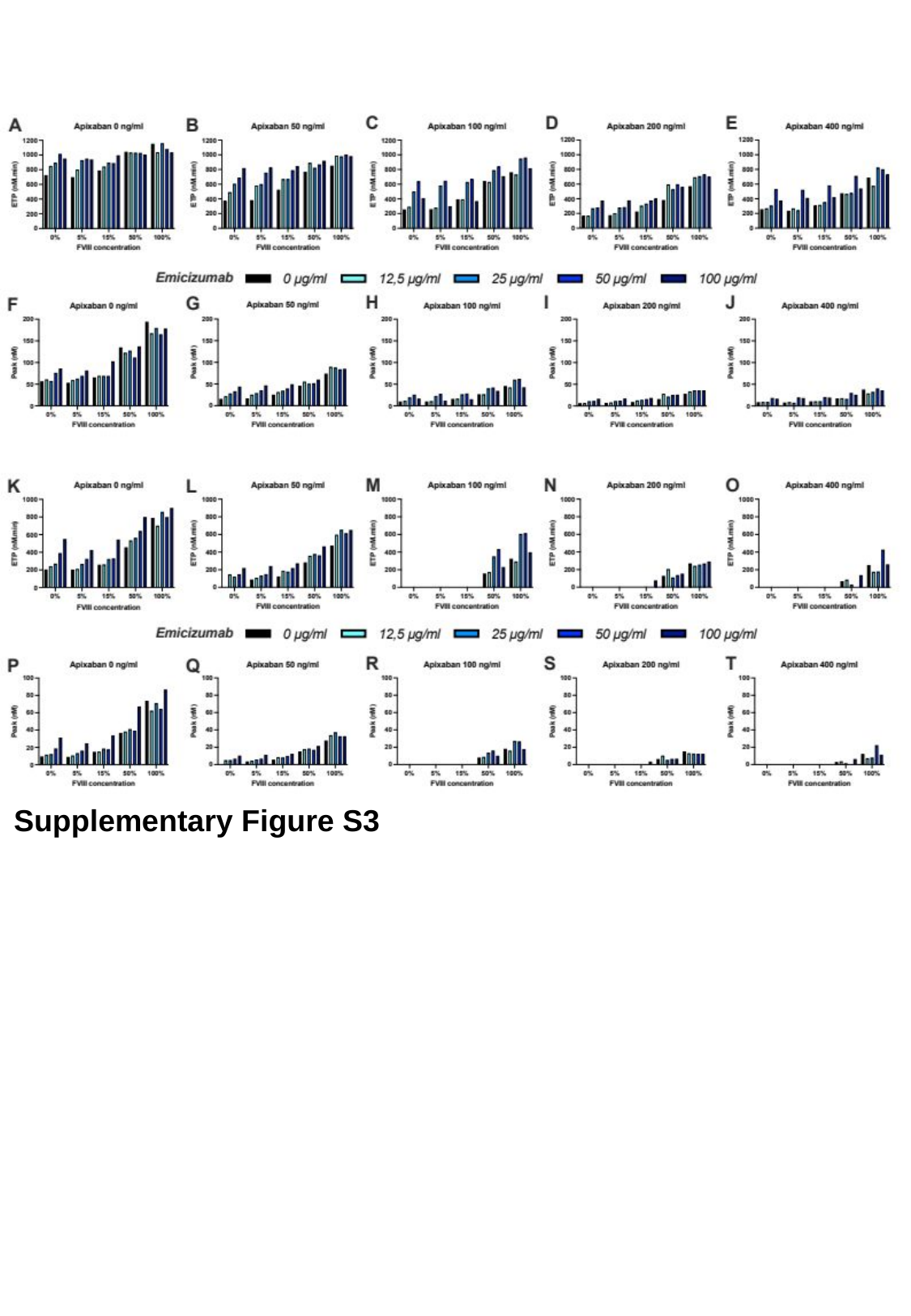

Supplementary Figure S3

## Slide 4
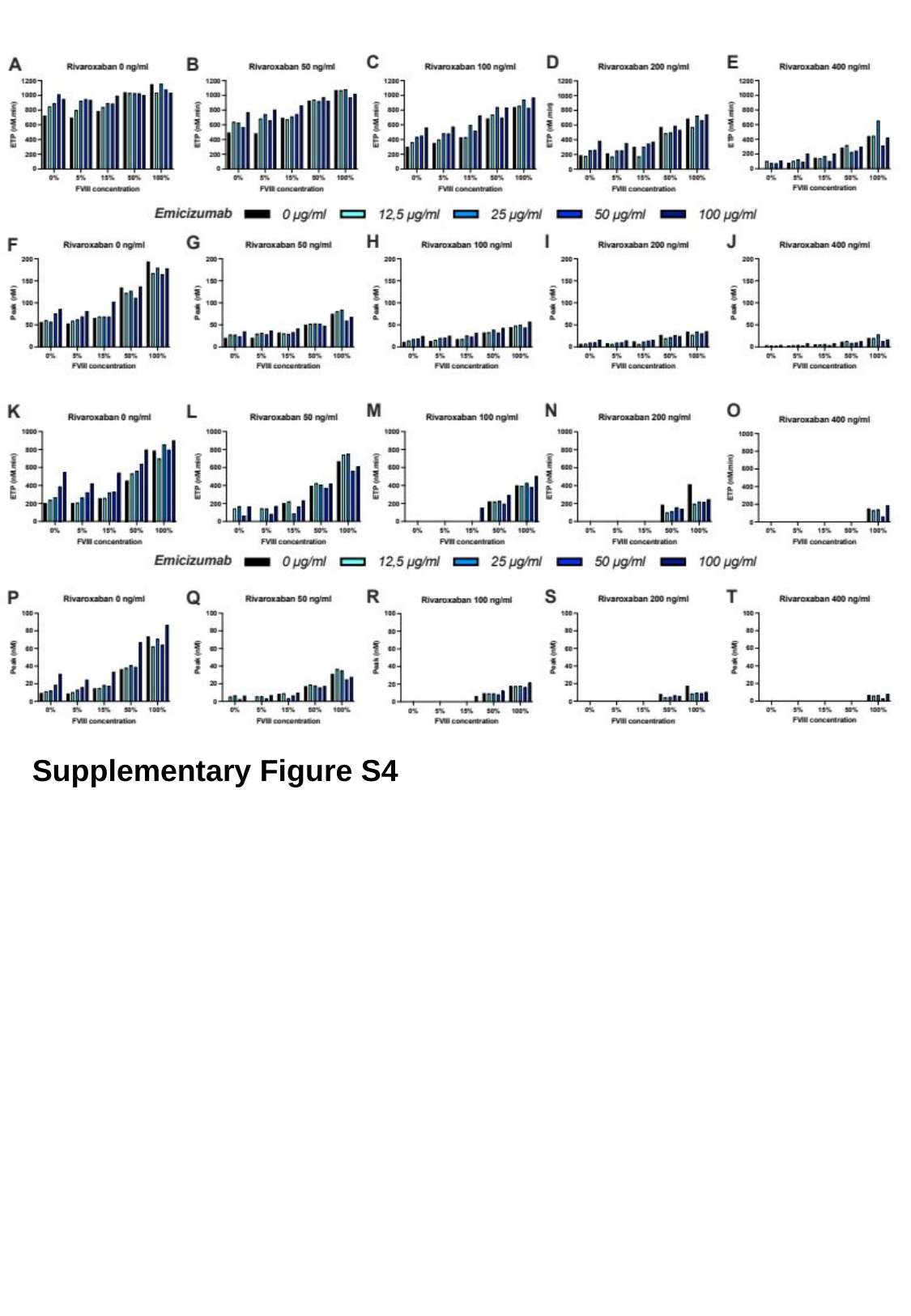

Supplementary Figure S4

## Slide 5
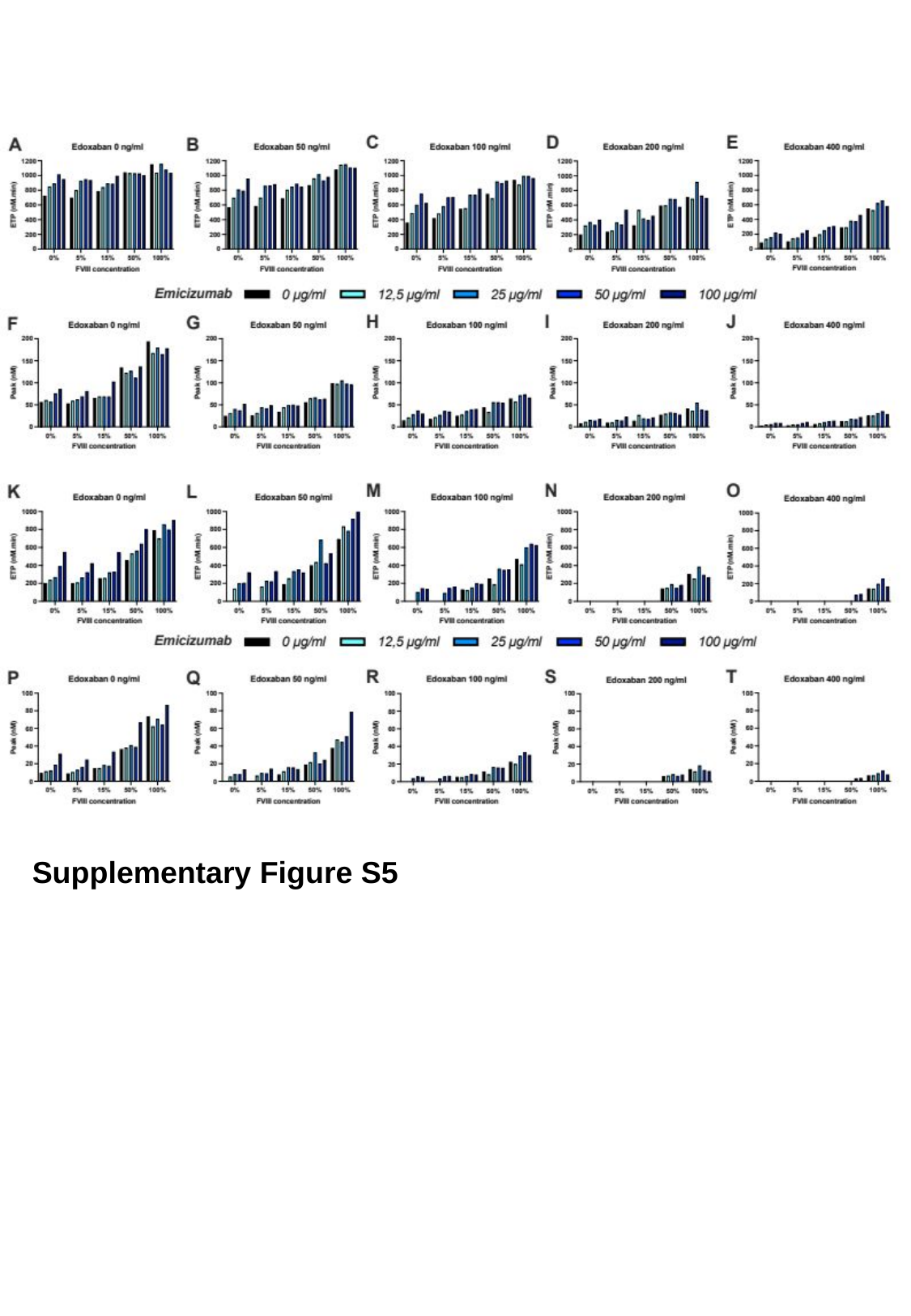

Supplementary Figure S5

## Slide 6
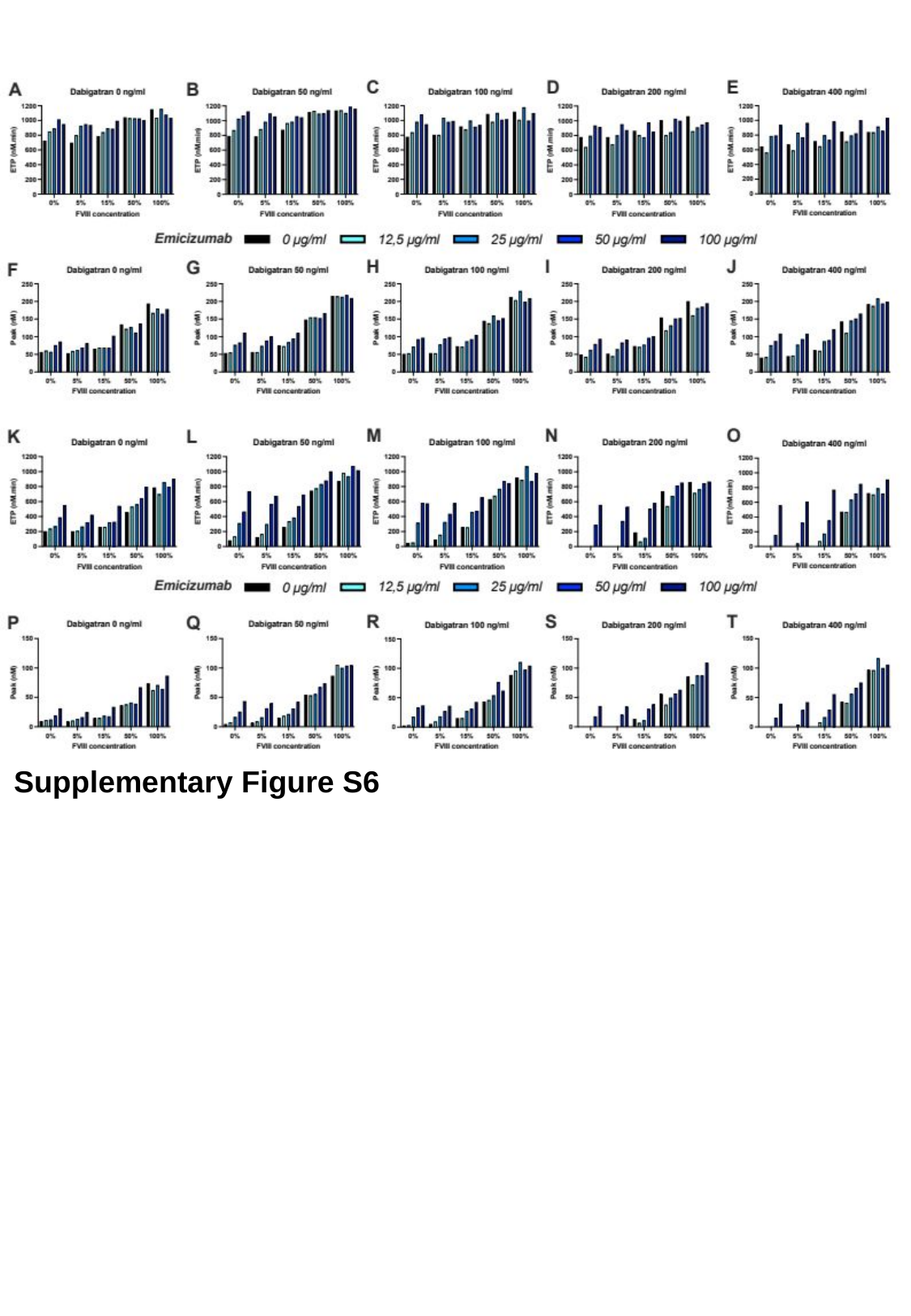

Supplementary Figure S6
