# Supplementary material for: Thrombin generation to evaluate the complex hemostatic balance of hemophilia A plasma containing direct oral anticoagulant and supplemented by factor VIII
Source: Res Pract Thromb Haemost. 2024 Sep 23;8(7):102576. doi: 10.1016/j.rpth.2024.102576 (PMC11532490; doi:10.1016/j.rpth.2024.102576)
Supplement: Supplementary Tables [file mmc2.docx]

**Supplementary Table 1. Coagulation parameters of a FVIII-deficient plasma.** Prothrombin time (PT) and activated partial thromboplastin time (aPTT) were evaluated in FVIII-deficient plasma aliquots supplemented or not with different doses of emicizumab and DOACs, as indicated.

|  |  | **Apixaban** | |  | **Rivaroxaban** | |  | **Edoxaban** | |  | **Dabigatran** | |
| --- | --- | --- | --- | --- | --- | --- | --- | --- | --- | --- | --- | --- |
| *Emicizumab*  *(µg/ml)* | *DOAC*  *(ng/ml)* | *PT (s)* | *aPTT (s)* |  | *PT (s)* | *aPTT (s)* |  | *PT (s)* | *aPTT (s)* |  | *PT (s)* | *aPTT (s)* |
| 0 | 0 | 14.3 | 97.7 |  | 14.3 | 97.7 |  | 14.3 | 97.7 |  | 14.3 | 97.7 |
|  | 50 | 14.9 | 74.3 |  | 15.8 | 116.9 |  | 16.2 | 95.6 |  | 15.9 | 180.0 |
|  | 100 | 15.2 | 108.8 |  | 17.1 | 132.5 |  | 17.1 | 123.0 |  | 15.7 | 168.0 |
|  | 200 | 16.6 | 114.5 |  | 18.9 | 118.1 |  | 22.0 | 139.8 |  | 17.0 | >180.0 |
|  | 400 | 16.3 | 119.6 |  | 23.7 | 154.0 |  | 29.9 | 169.2 |  | 19.2 | >180.0 |
| 12.5 | 0 | 14.3 | 29.7 |  | 14.3 | 29.7 |  | 14.3 | 29.7 |  | 14.3 | 29.7 |
|  | 50 | 15.2 | 30.7 |  | 14.9 | 30.6 |  | 16.2 | 33.2 |  | 16.0 | 37.5 |
|  | 100 | 15.6 | 32.1 |  | 17.1 | 33.5 |  | 17.0 | 34.1 |  | 16.8 | 39.2 |
|  | 200 | 17.2 | 33.6 |  | 20.0 | 33.5 |  | 21.5 | 38.0 |  | 19.4 | 47.3 |
|  | 400 | 17.1 | 33.4 |  | 22.7 | 38.8 |  | 28.7 | 42.9 |  | 21.1 | 49.6 |
| 25 | 0 | 14.5 | 27.9 |  | 14.5 | 27.9 |  | 14.5 | 27.9 |  | 14.5 | 27.9 |
|  | 50 | 15.0 | 27.8 |  | 15.2 | 28.4 |  | 16.0 | 30.6 |  | 15.8 | 33.1 |
|  | 100 | 15.5 | 28.7 |  | 17.0 | 30.2 |  | 17.5 | 31.4 |  | 16.3 | 34.2 |
|  | 200 | 16.9 | 29.9 |  | 20.0 | 33.0 |  | 21.1 | 35.3 |  | 18.8 | 41.1 |
|  | 400 | 18.4 | 31.7 |  | 24.2 | 36.0 |  | 29.7 | 40.7 |  | 19.2 | 38.3 |
| 50 | 0 | 14.6 | 26.9 |  | 14.6 | 26.9 |  | 14.6 | 26.9 |  | 14.6 | 26.9 |
|  | 50 | 15.2 | 26.9 |  | 15.8 | 27.4 |  | 16.2 | 28.4 |  | 16.1 | 31.5 |
|  | 100 | 15.6 | 27.9 |  | 17.8 | 28.9 |  | 17.2 | 29.1 |  | 16.5 | 32.7 |
|  | 200 | 16.7 | 28.8 |  | 20.6 | 31.2 |  | 21.6 | 33.1 |  | 17.1 | 34.0 |
|  | 400 | 16.9 | 28.9 |  | 29.0 | 37.1 |  | 29.2 | 38.0 |  | 29.2 | 38.5 |
| 100 | 0 | 14.7 | 25.1 |  | 14.7 | 25.1 |  | 14.7 | 25.1 |  | 14.7 | 25.1 |
|  | 50 | 15.1 | 25.6 |  | 15.8 | 26.5 |  | 16.3 | 27.9 |  | 16.3 | 29.5 |
|  | 100 | 15.6 | 26.5 |  | 17.6 | 28.2 |  | 18.1 | 29.3 |  | 17.4 | 33.4 |
|  | 200 | 16.6 | 27.3 |  | 20.7 | 30.5 |  | 20.9 | 31.2 |  | 17.4 | 31.2 |
|  | 400 | 17.0 | 27.5 |  | 29.7 | 36.1 |  | 30.8 | 37.2 |  | 18.4 | 37.2 |

**Supp Table 2. Coagulation parameters in a FVIII-deficient plasma.** Prothrombin time (PT) and activated partial thromboplastin time (aPTT) were evaluated in a FVIII-deficient plasma supplemented or not with different doses of emicizumab, DOACs and FVIII as indicated.

|  |  |  | **Apixaban** | |  | **Rivaroxaban** | |  | **Edoxaban** | |  | **Dabigatran** | |
| --- | --- | --- | --- | --- | --- | --- | --- | --- | --- | --- | --- | --- | --- |
| *Emicizumab*  *(µg/ml)* | *DOAC*  *(ng/ml)* | *Factor VIII*  *UI/dL* | *PT (s)* | *aPTT (s)* |  | *PT (s)* | *aPTT (s)* |  | *PT (s)* | *aPTT (s)* |  | *PT (s)* | *aPTT (s)* |
| 0 | 0 | 0 | 14.3 | 97.7 |  | 14.3 | 97.7 |  | 14.3 | 97.7 |  | 14.3 | 97.7 |
|  |  | 5 | 14.5 | 67.4 |  | 14.5 | 67.4 |  | 14.5 | 67.4 |  | 14.5 | 67.4 |
|  |  | 15 | 14.5 | 53.3 |  | 14.5 | 53.3 |  | 14.5 | 53.3 |  | 14.5 | 53.3 |
|  |  | 50 | 14.5 | 38.4 |  | 14.5 | 38.4 |  | 14.5 | 38.4 |  | 14.5 | 38.4 |
|  |  | 100 | 14.5 | 35.3 |  | 14.5 | 35.3 |  | 14.5 | 35.3 |  | 14.5 | 35.3 |
|  | 50 | 0 | 14.9 | 74.3 |  | 15.8 | 116.9 |  | 16.2 | 95.6 |  | 15.9 | 180.0 |
|  |  | 5 | 15.0 | 76.8 |  | 15.9 | 100.0 |  | 16.2 | 99.1 |  | 16.0 | 126.4 |
|  |  | 15 | 15.0 | 58.0 |  | 15.5 | 59.0 |  | 16.0 | 65.6 |  | 15.8 | 82.2 |
|  |  | 50 | 14.9 | 43.1 |  | 15.5 | 44.6 |  | 16.0 | 45.4 |  | 15.9 | 55.3 |
|  |  | 100 | 15.0 | 38.7 |  | 15.5 | 38.6 |  | 15.8 | 39.3 |  | 15.8 | 46.2 |
|  | 100 | 0 | 15.2 | 108.8 |  | 17.1 | 132.5 |  | 17.1 | 123.0 |  | 15.7 | 168.5 |
|  |  | 5 | 15.2 | 94.8 |  | 16.9 | 103.8 |  | 17.0 | 102.0 |  | 15.8 | 115.8 |
|  |  | 15 | 15.3 | 58.3 |  | 17.0 | 68.2 |  | 17.0 | 64.6 |  | 15.9 | 79.4 |
|  |  | 50 | 15.3 | 43.8 |  | 18.9 | 46.0 |  | 17.0 | 46.1 |  | 15.8 | 53.8 |
|  |  | 100 | 15.3 | 38.9 |  | 18.5 | 41.3 |  | 16.9 | 40.5 |  | 15.8 | 45.1 |
|  | 200 | 0 | 16.6 | 114.5 |  | 18.9 | 118.1 |  | 22.0 | 139.8 |  | 17.0 | >180.0 |
|  |  | 5 | 16.7 | 90.2 |  | 18.5 | 104.7 |  | 21.4 | 117.1 |  | 16.9 | 149.2 |
|  |  | 15 | 16.8 | 61.7 |  | 18.7 | 68.2 |  | 21.2 | 71.3 |  | 17.0 | 93.5 |
|  |  | 50 | 16.5 | 46.0 |  | 18.4 | 48.0 |  | 20.9 | 50.9 |  | 17.1 | 61.0 |
|  |  | 100 | 16.7 | 40.4 |  | 18.4 | 41.5 |  | 21.2 | 44.1 |  | 17.2 | 55.5 |
|  | 400 | 0 | 16.3 | 119.6 |  | 23.7 | 154.0 |  | 29.9 | 169.2 |  | 19.2 | >180.0 |
|  |  | 5 | 16.9 | 95.5 |  | 22.6 | 118.7 |  | 28.6 | 129.7 |  | 18.8 | 157.4 |
|  |  | 15 | 16.7 | 61.4 |  | 23.5 | 78.0 |  | 28.6 | 79.0 |  | 19.4 | 131.4 |
|  |  | 50 | 16.7 | 43.7 |  | 23.3 | 53.5 |  | 28.7 | 58.7 |  | 18.9 | 73.2 |
|  |  | 100 | 16.5 | 39.1 |  | 21.6 | 44.0 |  | 28.3 | 49.0 |  | 19.0 | 60.6 |

|  |  |  | **Apixaban** | |  | **Rivaroxaban** | |  | **Edoxaban** | |  | **Dabigatran** | | |
| --- | --- | --- | --- | --- | --- | --- | --- | --- | --- | --- | --- | --- | --- | --- |
| *Emicizumab*  *(µg/ml)* | *DOAC*  *(ng/ml)* | *Factor VIII*  *UI/dL* | *PT (s)* | *aPTT (s)* |  | *PT (s)* | *aPTT (s)* |  | *PT (s)* | *aPTT (s)* |  | *PT (s)* | *aPTT (s)* |  |
| 12.5 | 0 | 0 | 14.3 | 29.7 |  | 14.3 | 29.7 |  | 14.3 | 29.7 |  | 14.3 | 29.7 |  |
|  |  | 5 | 14.5 | 30.2 |  | 14.5 | 30.2 |  | 14.5 | 30.2 |  | 14.5 | 30.2 |  |
|  |  | 15 | 14.4 | 29.9 |  | 14.4 | 29.9 |  | 14.4 | 29.9 |  | 14.4 | 29.9 |  |
|  |  | 50 | 14.6 | 28.8 |  | 14.6 | 28.8 |  | 14.6 | 28.8 |  | 14.6 | 28.8 |  |
|  |  | 100 | 14.8 | 28.3 |  | 14.8 | 28.3 |  | 14.8 | 28.3 |  | 14.8 | 28.3 |  |
|  | 50 | 0 | 15.2 | 30.7 |  | 14.9 | 30.6 |  | 16.2 | 33.2 |  | 16.0 | 37.5 |  |
|  |  | 5 | 14.9 | 30.5 |  | 15.0 | 30.4 |  | 16.2 | 33.2 |  | 16.3 | 36.8 |  |
|  |  | 15 | 15.1 | 29.8 |  | 15.1 | 30.4 |  | 16.2 | 32.5 |  | 16.0 | 36.3 |  |
|  |  | 50 | 15.2 | 28.8 |  | 15.2 | 29.0 |  | 16.3 | 31.2 |  | 16.1 | 33.9 |  |
|  |  | 100 | 15.0 | 27.7 |  | 15.0 | 27.8 |  | 16.0 | 29.9 |  | 16.1 | 32.5 |  |
|  | 100 | 0 | 15.6 | 32.1 |  | 17.1 | 33.5 |  | 17.0 | 34.1 |  | 16.8 | 39.2 |  |
|  |  | 5 | 15.6 | 32.8 |  | 16.7 | 33.8 |  | 17.1 | 33.5 |  | 16.6 | 37.8 |  |
|  |  | 15 | 15.5 | 31.4 |  | 17.3 | 33.5 |  | 17.0 | 33.8 |  | 16.4 | 36.3 |  |
|  |  | 50 | 15.6 | 29.9 |  | 16.9 | 31.0 |  | 18.3 | 33.3 |  | 16.7 | 34.7 |  |
|  |  | 100 | 15.5 | 28.9 |  | 17.1 | 30.1 |  | 17.8 | 31.4 |  | 16.2 | 32.9 |  |
|  | 200 | 0 | 17.2 | 33.6 |  | 20.0 | 35.8 |  | 21.5 | 38.0 |  | 19.4 | 47.3 |  |
|  |  | 5 | 17.5 | 32.9 |  | 20.1 | 35.7 |  | 21.0 | 38.4 |  | 19.4 | 45.7 |  |
|  |  | 15 | 16.9 | 32.1 |  | 20.5 | 35.4 |  | 20.0 | 36.1 |  | 18.5 | 44.6 |  |
|  |  | 50 | 17.0 | 30.7 |  | 20.0 | 33.1 |  | 20.9 | 35.1 |  | 19.3 | 42.0 |  |
|  |  | 100 | 17.0 | 29.0 |  | 20.4 | 32.3 |  | 21.9 | 34.2 |  | 18.7 | 40.5 |  |
|  | 400 | 0 | 17.1 | 33.4 |  | 22.7 | 38.8 |  | 28.7 | 42.9 |  | 21.1 | 49.6 |  |
|  |  | 5 | 17.2 | 33.8 |  | 23.3 | 39.5 |  | 27.3 | 42.2 |  | 21.0 | 48.5 |  |
|  |  | 15 | 17.1 | 33.3 |  | 24.7 | 39.5 |  | 27.6 | 40.9 |  | 19.9 | 45.7 |  |
|  |  | 50 | 17.0 | 31.0 |  | 22.6 | 35.0 |  | 29.6 | 39.1 |  | 20.0 | 42.7 |  |
|  |  | 100 | 17.3 | 30.2 |  | 24.2 | 33.6 |  | 28.9 | 35.9 |  | 19.2 | 39.7 |  |

**Supp Table 2. *Continuation***

|  |  | |  | | **Apixaban** | | |  | | **Rivaroxaban** | | |  | **Edoxaban** | | |  | | **Dabigatran** | | |
| --- | --- | --- | --- | --- | --- | --- | --- | --- | --- | --- | --- | --- | --- | --- | --- | --- | --- | --- | --- | --- | --- |
| *Emicizumab*  *(µg/ml)* | | *DOAC*  *(ng/ml)* | | *Factor VIII*  *UI/dL* | | *PT (s)* | *aPTT (s)* | |  | | *PT (s)* | *aPTT (s)* |  | *PT (s)* | *aPTT (s)* |  | | *PT (s)* | | *aPTT (s)* |  |
| 25 | | 0 | | 0 | | 14.5 | 27.9 | |  | | 14.5 | 27.9 |  | 14.5 | 27.9 |  | | 14.5 | | 27.9 |  |
|  | |  | | 5 | | 14.6 | 28.1 | |  | | 14.6 | 28.1 |  | 14.6 | 28.1 |  | | 14.6 | | 28.1 |  |
|  | |  | | 15 | | 14.6 | 28.1 | |  | | 14.6 | 28.1 |  | 14.6 | 28.1 |  | | 14.6 | | 28.1 |  |
|  | |  | | 50 | | 14.7 | 27.5 | |  | | 14.7 | 27.5 |  | 14.7 | 27.5 |  | | 14.7 | | 27.5 |  |
|  | |  | | 100 | | 14.7 | 27.3 | |  | | 14.7 | 27.3 |  | 14.7 | 27.3 |  | | 14.7 | | 27.3 |  |
|  | | 50 | | 0 | | 15.0 | 27.8 | |  | | 15.2 | 28.4 |  | 16.0 | 30.6 |  | | 15.8 | | 33.1 |  |
|  | |  | | 5 | | 15.0 | 28.1 | |  | | 15.4 | 28.6 |  | 15.9 | 30.5 |  | | 15.9 | | 32.9 |  |
|  | |  | | 15 | | 15.0 | 27.7 | |  | | 15.8 | 28.6 |  | 15.9 | 29.8 |  | | 15.7 | | 32.3 |  |
|  | |  | | 50 | | 14.9 | 27.2 | |  | | 15.3 | 27.4 |  | 16.3 | 29.8 |  | | 15.9 | | 31.8 |  |
|  | |  | | 100 | | 15.0 | 26.4 | |  | | 15.1 | 26.2 |  | 15.8 | 28.3 |  | | 15.9 | | 30.7 |  |
|  | | 100 | | 0 | | 15.5 | 28.7 | |  | | 17.0 | 30.2 |  | 17.5 | 31.4 |  | | 16.3 | | 34.2 |  |
|  | |  | | 5 | | 15.6 | 29.0 | |  | | 16.8 | 29.7 |  | 17.4 | 31.3 |  | | 16.2 | | 34.1 |  |
|  | |  | | 15 | | 15.6 | 28.6 | |  | | 17.2 | 29.3 |  | 17.3 | 31.0 |  | | 16.4 | | 34.3 |  |
|  | |  | | 50 | | 15.5 | 28.0 | |  | | 16.9 | 28.4 |  | 17.2 | 29.5 |  | | 16.3 | | 33.0 |  |
|  | |  | | 100 | | 15.5 | 27.6 | |  | | 16.7 | 27.9 |  | 17.2 | 28.8 |  | | 16.3 | | 32.7 |  |
|  | | 200 | | 0 | | 16.9 | 29.9 | |  | | 20.0 | 33.0 |  | 21.1 | 35.3 |  | | 18.8 | | 41.1 |  |
|  | |  | | 5 | | 16.8 | 30.3 | |  | | 20.1 | 32.7 |  | 20.9 | 34.7 |  | | 18.4 | | 40.4 |  |
|  | |  | | 15 | | 16.8 | 29.7 | |  | | 21.2 | 33.5 |  | 22.8 | 34.5 |  | | 18.8 | | 40.8 |  |
|  | |  | | 50 | | 16.9 | 29.4 | |  | | 19.9 | 31.4 |  | 20.6 | 32.9 |  | | 18.6 | | 38.6 |  |
|  | |  | | 100 | | 16.8 | 28.1 | |  | | 20.0 | 30.6 |  | 20.5 | 31.4 |  | | 18.1 | | 37.6 |  |
|  | | 400 | | 0 | | 18.4 | 31.7 | |  | | 24.2 | 36.0 |  | 29.7 | 40.7 |  | | 19.2 | | 38.3 |  |
|  | |  | | 5 | | 18.5 | 31.9 | |  | | 23.5 | 34.8 |  | 29.0 | 40.4 |  | | 19.6 | | 39.8 |  |
|  | |  | | 15 | | 18.3 | 31.1 | |  | | 23.4 | 34.6 |  | 29.1 | 39.8 |  | | 19.4 | | 41.2 |  |
|  | |  | | 50 | | 18.1 | 30.1 | |  | | 27.3 | 35.0 |  | 29.1 | 37.9 |  | | 19.0 | | 41.4 |  |
|  | |  | | 100 | | 18.1 | 29.1 | |  | | 23.0 | 31.4 |  | 28.2 | 35.7 |  | | 19.3 | | 41.7 |  |

**Supp Table 2. *Continuation***

|  |  | |  | | **Apixaban** | | |  | | **Rivaroxaban** | | |  | **Edoxaban** | | |  | | **Dabigatran** | | |
| --- | --- | --- | --- | --- | --- | --- | --- | --- | --- | --- | --- | --- | --- | --- | --- | --- | --- | --- | --- | --- | --- |
| *Emicizumab*  *(µg.ml)* | | *DOAC*  *(ng.ml)* | | *Factor VIII*  *UI/dL* | | *PT (s)* | *aPTT (s)* | |  | | *PT (s)* | *aPTT (s)* |  | *PT (s)* | *aPTT (s)* |  | | *PT (s)* | | *aPTT (s)* |  |
| 50 | | 0 | | 0 | | 14.6 | 26.9 | |  | | 14.6 | 26.9 |  | 14.6 | 26.9 |  | | 14.6 | | 26.9 |  |
|  | |  | | 5 | | 14.9 | 27.2 | |  | | 14.9 | 27.2 |  | 14.9 | 27.2 |  | | 14.9 | | 27.2 |  |
|  | |  | | 15 | | 15.0 | 26.9 | |  | | 15.0 | 26.9 |  | 15.0 | 26.9 |  | | 15.0 | | 26.9 |  |
|  | |  | | 50 | | 15.0 | 27.3 | |  | | 15.0 | 27.3 |  | 15.0 | 27.3 |  | | 15.0 | | 27.3 |  |
|  | |  | | 100 | | 14.9 | 26.6 | |  | | 14.9 | 26.6 |  | 14.9 | 26.6 |  | | 14.9 | | 26.6 |  |
|  | | 50 | | 0 | | 15.2 | 26.9 | |  | | 15.8 | 27.4 |  | 16.2 | 28.4 |  | | 16.1 | | 31.5 |  |
|  | |  | | 5 | | 15.3 | 26.8 | |  | | 15.9 | 27.6 |  | 16.0 | 28.7 |  | | 16.0 | | 30.6 |  |
|  | |  | | 15 | | 15.1 | 26.7 | |  | | 16.3 | 27.8 |  | 16.1 | 28.4 |  | | 16.1 | | 30.5 |  |
|  | |  | | 50 | | 15.5 | 26.4 | |  | | 15.6 | 26.9 |  | 16.3 | 28.7 |  | | 15.9 | | 30.1 |  |
|  | |  | | 100 | | 15.1 | 25.9 | |  | | 15.9 | 26.8 |  | 16.0 | 28.1 |  | | 16.0 | | 29.6 |  |
|  | | 100 | | 0 | | 15.6 | 27.9 | |  | | 17.8 | 28.9 |  | 17.2 | 29.1 |  | | 16.5 | | 32.7 |  |
|  | |  | | 5 | | 15.8 | 27.8 | |  | | 17.4 | 28.4 |  | 17.4 | 29.2 |  | | 17.6 | | 35.1 |  |
|  | |  | | 15 | | 15.8 | 28.1 | |  | | 17.6 | 28.5 |  | 17.3 | 29.2 |  | | 17.7 | | 35.6 |  |
|  | |  | | 50 | | 15.7 | 27.6 | |  | | 17.6 | 28.4 |  | 17.4 | 28.7 |  | | 17.5 | | 34.6 |  |
|  | |  | | 100 | | 15.6 | 26.9 | |  | | 17.4 | 27.9 |  | 17.7 | 28.5 |  | | 17.7 | | 34.5 |  |
|  | | 200 | | 0 | | 16.7 | 28.8 | |  | | 20.6 | 31.2 |  | 21.6 | 33.1 |  | | 17.1 | | 34.0 |  |
|  | |  | | 5 | | 16.8 | 28.7 | |  | | 20.5 | 31.4 |  | 21.4 | 32.5 |  | | 16.9 | | 33.7 |  |
|  | |  | | 15 | | 16.9 | 28.4 | |  | | 20.3 | 31.0 |  | 21.4 | 32.4 |  | | 16.9 | | 33.4 |  |
|  | |  | | 50 | | 16.1 | 27.2 | |  | | 20.3 | 30.3 |  | 20.8 | 31.4 |  | | 17.0 | | 32.9 |  |
|  | |  | | 100 | | 16.4 | 27.1 | |  | | 20.6 | 29.8 |  | 20.4 | 30.4 |  | | 17.5 | | 32.9 |  |
|  | | 400 | | 0 | | 16.9 | 28.9 | |  | | 29.0 | 37.1 |  | 29.2 | 38.0 |  | | 20.0 | | 38.5 |  |
|  | |  | | 5 | | 16.8 | 28.9 | |  | | 27.5 | 36.4 |  | 27.6 | 37.7 |  | | 19.8 | | 38.9 |  |
|  | |  | | 15 | | 17.3 | 29.5 | |  | | 29.3 | 36.5 |  | 27.4 | 36.9 |  | | 19.9 | | 39.9 |  |
|  | |  | | 50 | | 17.1 | 28.9 | |  | | 26.8 | 35.0 |  | 27.4 | 36.4 |  | | 19.8 | | 40.2 |  |
|  | |  | | 100 | | 16.9 | 28.2 | |  | | 28.5 | 34.6 |  | 26.6 | 34.0 |  | | 19.5 | | 39.8 |  |

**Supp Table 2. *Continuation***

|  |  | | |  | | **Apixaban** | |  | | **Rivaroxaban** | | |  | | **Edoxaban** | | |  | | **Dabigatran** | |  |
| --- | --- | --- | --- | --- | --- | --- | --- | --- | --- | --- | --- | --- | --- | --- | --- | --- | --- | --- | --- | --- | --- | --- |
| *Emicizumab*  *(µg/ml)* | | *DOAC*  *(ng/ml)* | *Factor VIII*  *UI/dL* | | *PT (s)* | | *aPTT (s)* |  | *PT (s)* | | *aPTT (s)* |  | | *PT (s)* | | *aPTT (s)* |  | | *PT (s)* | | *aPTT (s)* | |
| 100 | | 0 | 0 | | 14.7 | | 25.1 |  | 14.7 | | 25.1 |  | | 14.7 | | 25.1 |  | | 14.7 | | 25.1 | |
|  | |  | 5 | | 14.9 | | 25.7 |  | 14.9 | | 25.7 |  | | 14.9 | | 25.7 |  | | 14.9 | | 25.7 | |
|  | |  | 15 | | 15.0 | | 25.2 |  | 15.0 | | 25.2 |  | | 15.0 | | 25.2 |  | | 15.0 | | 25.2 | |
|  | |  | 50 | | 14.9 | | 25.2 |  | 14.9 | | 25.2 |  | | 14.9 | | 25.2 |  | | 14.9 | | 25.2 | |
|  | |  | 100 | | 14.9 | | 25.4 |  | 14.9 | | 25.4 |  | | 14.9 | | 25.4 |  | | 14.9 | | 25.4 | |
|  | | 50 | 0 | | 15.1 | | 25.6 |  | 15.8 | | 26.5 |  | | 16.3 | | 27.9 |  | | 16.3 | | 29.5 | |
|  | |  | 5 | | 15.1 | | 25.5 |  | 16.0 | | 26.4 |  | | 16.4 | | 27.9 |  | | 16.2 | | 29.7 | |
|  | |  | 15 | | 15.2 | | 25.6 |  | 15.7 | | 26.1 |  | | 16.5 | | 28.4 |  | | 16.0 | | 29.6 | |
|  | |  | 50 | | 15.2 | | 25.1 |  | 15.9 | | 26.0 |  | | 16.5 | | 28.0 |  | | 15.9 | | 29.0 | |
|  | |  | 100 | | 15.2 | | 25.2 |  | 15.7 | | 25.8 |  | | 16.3 | | 27.5 |  | | 15.9 | | 28.8 | |
|  | | 100 | 0 | | 15.6 | | 26.5 |  | 17.6 | | 28.2 |  | | 18.1 | | 29.3 |  | | 17.4 | | 33.4 | |
|  | |  | 5 | | 16.0 | | 27.1 |  | 17.8 | | 27.9 |  | | 18.2 | | 29.2 |  | | 17.5 | | 33.8 | |
|  | |  | 15 | | 15.6 | | 26.9 |  | 17.6 | | 27.8 |  | | 18.2 | | 29.0 |  | | 17.4 | | 33.5 | |
|  | |  | 50 | | 15.7 | | 27.1 |  | 17.5 | | 27.7 |  | | 17.8 | | 28.9 |  | | 17.1 | | 32.4 | |
|  | |  | 100 | | 15.7 | | 27.0 |  | 17.6 | | 27.4 |  | | 18.0 | | 28.4 |  | | 17.0 | | 32.1 | |
|  | | 200 | 0 | | 16.6 | | 27.3 |  | 20.7 | | 30.5 |  | | 20.9 | | 31.2 |  | | 17.4 | | 32.9 | |
|  | |  | 5 | | 16.5 | | 27.7 |  | 20.7 | | 30.3 |  | | 20.4 | | 30.5 |  | | 17.6 | | 33.0 | |
|  | |  | 15 | | 16.5 | | 27.4 |  | 20.3 | | 30.2 |  | | 20.0 | | 30.5 |  | | 18.1 | | 34.4 | |
|  | |  | 50 | | 16.6 | | 27.1 |  | 20.9 | | 29.5 |  | | 20.9 | | 30.5 |  | | 17.1 | | 31.7 | |
|  | |  | 100 | | 16.4 | | 26.1 |  | 20.7 | | 29.7 |  | | 21.1 | | 30.5 |  | | 17.4 | | 32.3 | |
|  | | 400 | 0 | | 17.0 | | 27.5 |  | 29.7 | | 36.1 |  | | 30.8 | | 37.2 |  | | 18.4 | | 35.7 | |
|  | |  | 5 | | 16.9 | | 27.8 |  | 26.5 | | 33.3 |  | | 30.3 | | 37.6 |  | | 18.2 | | 35.1 | |
|  | |  | 15 | | 17.2 | | 28.0 |  | 27.5 | | 34.8 |  | | 29.8 | | 36.9 |  | | 18.0 | | 34.9 | |
|  | |  | 50 | | 16.9 | | 27.6 |  | 27.3 | | 32.2 |  | | 29.1 | | 36.2 |  | | 18.1 | | 34.9 | |
|  | |  | 100 | | 17.0 | | 27.7 |  | 27.3 | | 33.3 |  | | 30.3 | | 35.1 |  | | 18.7 | | 34.9 | |

**Supp Table 2. *Continuation***
